# Mitogen-Activated Protein Kinase CsPMK1 Is Essential for Pepper Fruit Anthracnose by *Colletotrichum scovillei*

**DOI:** 10.3389/fmicb.2022.770119

**Published:** 2022-02-24

**Authors:** Teng Fu, Jong-Hwan Shin, Noh-Hyun Lee, Kwang Ho Lee, Kyoung Su Kim

**Affiliations:** Division of Bio-Resource Sciences, BioHerb Research Institute, and Interdisciplinary Program in Smart Agriculture, Kangwon National University, Chuncheon, South Korea

**Keywords:** *Colletotrichum scovillei*, mitogen-activated protein kinase, pepper fruit, anthracnose, stress adaptation, homeobox

## Abstract

The phytopathogenic fungus *Colletotrichum scovillei*, belonging to the *Colletotrichum acutatum* species complex, causes severe anthracnose disease on several fruits, including chili pepper (*Capsicum annuum*). However, the molecular mechanisms underlying the development and pathogenicity of *Colletotrichum scovillei* are unclear. The conserved Fus3/Kss1-related MAPK regulates fungal development and pathogenicity. Here, the role of *CsPMK1*, orthologous to *Fus3/Kss1*, was characterized by phenotypic comparison of a target deletion mutant (*ΔCspmk1*). The mycelial growth and conidiation of *ΔCspmk1* were normal compared to that of the wild type. *ΔCspmk1* produced morphologically abnormal conidia, which were delayed in conidial germination. Germinated conidia of *ΔCspmk1* failed to develop appressoria on inductive surfaces of hydrophobic coverslips and host plants. *ΔCspmk1* was completely defective in infectious growth, which may result from failure to suppress host immunity. Furthermore, *ΔCspmk1* was impaired in nuclear division and lipid mobilization during appressorium formation, in response to a hydrophobic surface. CsPMK1 was found to interact with CsHOX7, a homeobox transcription factor essential for appressorium formation, *via* a yeast two-hybridization analysis. Taken together, these findings suggest that *CsPMK1* is required for fungal development, stress adaptation, and pathogenicity of *C. scovillei*.

## Introduction

Fungal species of the genus *Colletotrichum* cause serious anthracnose disease on a wide range of crops, vegetables, and fruits worldwide, resulting in considerable economic losses (Perfect et al., 1999; Cannon et al., 2012; De Silva et al., 2019). To date, most research has focused on foliar diseases, whereas the development of anthracnose on fruit is unclear (Epstein et al., 1998; Fukada and Kubo, 2015; Fang et al., 2018; Yang et al., 2018). It is therefore of great interest to study the molecular mechanisms underlying the development of fruit anthracnose disease. *Colletotrichum scovillei*, belonging to the *Colletotrichum acutatum* species complex, has been reported to cause severe anthracnose disease on pepper fruits in tropical and temperate zones (Caires et al., 2014; Kanto et al., 2014; Zhao et al., 2016; Oo et al., 2017; Noor and Zakaria, 2018; De Silva et al., 2019; Khalimi et al., 2019). Similar to other plant pathogenic fungi, *C. scovillei* propagates conidiation by producing a large number of asexual spores (Fu et al., 2021). Infection of *C. scovillei* starts when conidia attach to the surface of pepper fruits. Upon recognition of host signals, conidia emerge to germinate and differentiate an appressorium from the tip of the germ tube, which subsequently penetrates the host cuticle layer. During appressorium-mediated penetration, a unique dendroid structure appears in the cuticle layer of pepper fruits (Fu et al., 2021). Following successful colonization in epidermal cells, the fungus causes typical sunken anthracnose lesions with a large amount of pinkish conidia, which contributes to the next round of infection (Fu et al., 2021).

Plant pathogenic fungi have evolved sophisticated signal transduction networks in response to various environmental stimuli (Nair et al., 2019). Extracellular signals are sensed and recognized by cell membrane-integrated receptors, which trigger distinct effector targets, such as enzymes, small GTPases, and ion channels, thus activating downstream signaling pathways (Neves et al., 2002). An amount of work from different groups of model systems have revealed that the mitogen-activated protein kinase (MAPK) signaling pathway regulates multiple processes, including cell survival, differentiation, stress tolerance, and interactions with environmental factors (Avruch, 2007; Alonso-Monge et al., 2009; Saito, 2010; Martínez-Soto and Ruiz-Herrera, 2017). The MAPKs are a group of serine/threonine kinases, activated by external stimuli *via* a sequential MAPKKK-MAPKK-MAPK cascade, by which phosphorylation sites of tyrosine/threonine deliver signals (Cargnello and Roux, 2011; Liang et al., 2019). The model organism *Saccharomyces cerevisiae* has five MAPKs (Fus3, Kss1, Slt2, Hog1, and Smk1), which have been functionally characterized (Posas et al., 1998; Waltermann and Klipp, 2010).

The genomes of most plant pathogenic fungi contain three MAPKs, orthologous to Hog1, Fus3/Kss1, and Slt2 (Jiang et al., 2018). Studies in a number of evolutionarily distant plant pathogenic fungi revealed that the Fus3/Kss1 MAPK is essential for fungal pathogenicity (Xu and Hamer, 1996; Takano et al., 2000; Zheng et al., 2000; Di Pietro et al., 2001; Urban et al., 2003; Rauyaree et al., 2005; He et al., 2017; Liang et al., 2019), and for this reason, Fus/Kss1 MAPK is often referred to the pathogenicity MAP kinase 1 (PMK1). The orthologs of PMK1 were demonstrated to be essential for appressorium formation in appressorium-forming fungi, including the rice blast fungus *Magnaporthe oryzae*, the cucumber anthracnose fungus *Colletotrichum lagenarium*, and poplar anthracnose fungus *Colletotrichum gloeosporioides* (Xu and Hamer, 1996; Takano et al., 2000; He et al., 2017). Moreover, this MAPK is indispensable in plant penetration and infectious growth in many non-appressorium-forming fungi, such as the biotrophic fungus *Claviceps purpurea* and necrotrophic fungus *Fusarium graminearum* (Mey et al., 2002; Urban et al., 2003). In addition to regulatory role of PMK1 in fungal pathogenicity, several genes acting upstream (SHO1 and MSB2) and downstream (Ste12) of the PMK1-type MAPK signaling pathway have been characterized in *M. oryzae* (Liu et al., 1994, 2011; Park et al., 2002; So et al., 2018). The *MoMSB2* mutant was dramatically reduced in appressorium formation and virulence, and the *MoSHO1* mutant was slightly reduced in pathogenicity (Liu et al., 2011). Deletion of *Mst12*, orthologous to *Ste12*, did not affect appressorium formation but abolished plant penetration and infectious growth (Park et al., 2002). *PMK1* gene also regulates other aspects of fungal growth and development (Jiang et al., 2018). For example, *PMK1* is important for mycelial growth in *Colletotrichum fructicola* (Liang et al., 2019), conidiation in *Alternaria brassicicola* (Cho et al., 2007), conidial germination in *C. largenarium* (Takano et al., 2000), and conidium viability in *Aspergillus nidulans* (Kang et al., 2013). Finally, PMK1 regulates fumonisin biosynthesis in *Fusarium verticillioides* (Zhang et al., 2011), and glycogen and lipid metabolism in *M. oryzae* (Thines et al., 2000).

To investigate molecular mechanisms involved in anthracnose disease on pepper fruits, we have firstly decided to characterize a conserved upstream regulator, Fus3/Kss1 MAPK, in the development and pathogenicity of the pepper fruit anthracnose fungus *C. scovillei*. We generated target deletion mutant of *CsPMK1* and performed microscopic observation and phenotypic analysis. Our results showed that deletion of *CsPMK1* did not affect mycelial growth and conidiation but caused impairments in morphology and germination of conidium, and defects in tolerance to stress conditions. The *CsPMK1* deletion mutant failed to from appressorium and suppress host defense, thus resulting in abolishment of causing anthracnose disease. Based on a yeast two-hybridization analysis, CsPMK1 may interact with CsHOX7, a transcription factor essential for appressorium formation. The lipids metabolism and nuclear division were defective in the *CsPMK1* deletion mutant during appressorium developed from germ tube and mycelia. These results suggest that *CsPMK1* plays important roles in fungal developments, stress adaptation, and pathogenicity in *C. scovillei*. Our study provides a fundamental insight into mechanism underlying fruit anthracnose development, which would contribute to developing a novel strategy to control anthracnose disease of fruits.

## Materials and Methods

### Fungal Strains, Culture Conditions, and Nucleic Acid Isolation

The *C. scovillei* wild-type strain KC05 and its transformants were grown routinely at 25°C under continuous fluorescent light on minimal medium agar (MMA; 0.1 ml L^−1^ trace elements, 30 g L^−1^ sucrose, 2 g L^−1^ NaNO_3_, 1 g L^−1^ KH_2_PO_4_, 0.5 g L^−1^ KCl, 0.5 g L^−1^ MgSO_4_∙7H_2_O, and 15 g L^−1^ agar powder), V8 agar (V8A; 8% V8 juice and 1.5% agar powder), potato dextrose agar (PDA; 3.9% powder), or oatmeal agar (OMA; 5% oatmeal and 1.5% agar powder), as described previously (Fu et al., 2021). Mycelia cultured in liquid complete medium (CM; 0.06% yeast extract, 0.06% casamino acids, and 1% sucrose) and solid TB3 (TB3; 20% sucrose, 1% glucose, 0.3% yeast extract, 0.3% casamino acids, and 0.8% agar powder) were used for extraction of DNA or RNA (Shin et al., 2019). The fungal genomic DNA used for screening PCR and Southern blot was extracted by a rapid and safe method, and the standard method, respectively (Han et al., 2018). Total RNA was isolated from fungal tissues with liquid nitrogen using the Easy-Spin™ Total RNA Extraction Kit (iNtRON Biotechnology, Seongnam, South Korea), following the manufacturer’s instructions.

### Phylogenetic Analysis, Targeted Gene Deletion, and Complementation

Using the InterPro term IPR003527 (MAP kinase and conserved site) as a reference to search the genome of *C. scovillei*, three genetic loci were identified as: *CAP_011033*, *CAP_001359*, and *CAP_009200*.^1^ The phylogenetic relationship of MAPKs in *C. scovillei* with their orthologs in other fungi was analyzed using the maximum-likelihood method with 1,000 bootstraps in the MEGA 7 software (Fu et al., 2019). Targeted deletion of *CsPMK1* was performed by using a modified double-joint PCR (Yu et al., 2004). Fragments (1.5 kb in length) of the 5′- and 3′-flanking were amplified with the primers 5F/5R and 3F/3R (Supplementary Table S1), respectively. The two amplified fragments and the hygromycin B phosphotransferase (HPH) cassette, amplified with the primers HPHF/HPHR (Supplementary Table S1) from pBCATPH, were fused by fusion PCR (Han et al., 2015). This fused construct was next amplified by nested PCR with NF/NR (Supplementary Table S1) to generate gene-deletion constructs, which were transformed into wild-type protoplasts *via* a polyethylene glycol-mediated transformation method (Shin et al., 2019). The resulting transformants were firstly selected by screening PCR with primers SF/SR (Supplementary Table S1) and verified by Southern blot and reverse transcriptase-polymerase chain reaction (RT-PCR.). To generate complemented strains, genomic copies of the target gene, amplified with the primers CF/CR (Supplementary Table S1), were co-reintroduced into protoplasts of target gene-deletion mutant with geneticin-resistance cassette from pII99 (Yi et al., 2009). Transformants of the complemented strain were selected by screening PCR with the primers SF/SR (Supplementary Table S1) and confirmed by RT-PCR.

### Southern Blot and RT-PCR

Southern blot was performed as described previously (Kim et al., 2009; Han et al., 2015). Briefly, a DNA probe (500 bp in length), amplified with the primers PF/PR (Supplementary Table S1) from the wild-type genome, was labeled with Biotin-High Prime (Roche, United States). Genomic DNA isolated from the wild-type and candidate mutants were digested with restriction enzymes and transferred to a nylon hybridization membrane. The probe-labeled membrane was detected using the ChemiDoc XRS + System (Bio-Rad, United States). For RT-PCR, first-strand complementary DNA (cDNA) was synthesized using the SuperScript® III First-strand Synthesis System (Invitrogen, United States) from total RNA (5 μg), isolated from fungal tissues and plant tissues using the Easy-Spin™ Total RNA Extraction Kit (iNtRON Biotechnology, South Korea). The RT-PCR reaction comprised 10 ng of cDNA, 10 pmol of each primer, and 4 μl of Pfu Plus 5 × PCR Master Mix (Elpis, South Korea) and was run for 30 cycles in an Applied Biosystems Thermal Cycler. The mixtures of qRT-PCR reactions contained 15 ng of cDNA, 10 pmol of each primer of qrtF/qrtR primers (Supplementary Table S1), and 5 μl of Real-Time PCR 2× Master Mix (Elpis, South Korea). The PCR program was set as: 1 cycle (95°C for 3 min), followed by 40 cycles (95°C for 15 s, 58°C for 30 s, and 72°C for 30 s).

### Phenotypic Characterization of Mutants

Mycelial growth was evaluated by measuring colony diameters on complete medium agar (CMA) and MMA. Stress on mycelia was induced by growing the wild-type strain and transformants on CMA containing stress agents. Conidiation was assessed by counting conidia collected with 5 ml of distilled water from V8 agar, which had been incubated at 25°C in dark and 2 days in light. Conidial germination and appressorium formation were performed by dropping conidial suspension (20 μl; 5 × 10^4^ ml^−1^) on the hydrophobic surface of coverslips, which were placed in a humid plastic box, and incubated at 25°C. The numbers of germinated conidia and conidia with appressoria were counted in 100 conidia. To observe nuclear division and lipid mobilization, conidial suspensions were placed into the hydrophobic surface of coverslips. At predetermined time points during conidial germination and appressorium formation, DAPI and Nile red were used to stain nuclei and lipid droplets, respectively (Han et al., 2015). Conidium viability was evaluated by incubating conidia at 25°C and 37°C for 16 h, and staining with phloxine B. For anthracnose formation assays, conidial suspensions (10^6^ ml^−1^) were inoculated into wounded and intact pepper fruits and were incubated in a humid box for 6 and 9 days, respectively. Invasive hyphae in heat-killed host tissues were stained as described previously (Fu et al., 2021). Thin segments of infected tissues were sliced using a razor and immersed in fixation solution [60% (*v*/*v*) methanol, 30% (*v*/*v*) chloroform, and 10% (*v*/*v*) acetic acid]. The fixed samples were rehydrated in a series of ethanol solutions of decreasing concentrations and subsequently stained with modified trypan blue. Phenotypic analysis involved at least three independent experiments with three replicates per experiment. The significance of differences was analyzed using Duncan’s test (*p* < 0.05).

### Scanning Electron Microscope Analysis

Conidia suspension (5 × 10^4^ ml^−1^) was inoculated into intact pepper fruits. After 36 h, samples were sliced from infected plant tissues and fixed in 4% paraformaldehyde at 4°C for 24 h. After fixation, the samples were washed with 1 × PBS buffer. Subsequently, the samples were firstly dehydrated in a series of ethanol solutions of increasing concentrations (30, 40, 50, 60, 70, 80, 90, and 95%) for 1 h at each concentration, finally dehydrated in 100% ethanol for 1 h in three times (Pariona et al., 2019). The dehydrated samples were dried using critical-point dried method, mounted on the carbon tape, and coated with gold as described previously (Schroeckh et al., 2009). To observe penetration peg indentation, appressoria was detached from the surface of pepper fruit by sonication. The samples were observed using a scanning electron microscope (Analytical HR-SEM).

### Generation of GFP Fusion Constructs

The CsPMK1:sGFP vector was generated by overlap cloning (Han et al., 2018). Segments including the promoter region (1.5 kb) and ORF of the *CsPMK1* were amplified with the primers CsPMK1_F/CsPMK1_R (Supplementary Table S1), from wild-type genomic DNA. The sGFP (5.0 kb) fragment containing geneticin-resistance gene was amplified with primers pIG-CsPMK1_F/pIG-CsPMK1_R (Supplementary Table S1) from pIGPAPA-sGFP. The CsPMK1 fragment was fused with the sGFP fragment under the control of the native promoter. Recombinant CsPMK1:sGFP constructs were transformed into protoplasts of transformants expressing histone H1:DsRed (Fu et al., 2021). Fluorescence signals were detected by fluorescence microscopy (Carl Zeiss Microscope Division, Germany).

### Yeast Two-Hybridization Assay

The Matchmaker Gold Yeast Two-Hybrid System (Clontech) was used to assess the interaction between CsPMK1 and CsHOX7. cDNA of *CsPMK1* was amplified using primers p-PMK1_F/p-PMK1_R (Supplementary Table S1) and cloned into the prey vector pGADT7 (Clontech). Similarly, cDNA of CsHOX7 was amplified with primers p-HOX7_F/p-HOX7_R (Supplementary Table S1) and cloned into the bait vector pGBKT7 (Clontech). The recombinant plasmids were confirmed by sequencing. The plasmids used as positive and negative controls were supplied in the Matchmaker Gold Yeast Two-Hybrid System kit. The confirmed vectors (prey and bait) were co-transformed into Y2H Gold strain competent cells (Clontech), according to the manufacturer’s instructions. Yeast strains containing both prey and bait were selected on double dropout agar medium (SD-Leu-Trp). The selected yeast strains were then screened on quadruple dropout agar medium (SD-Leu-Trp-His-Ade/X-α-Gal/Aureobasidin A).

## Results

### Phylogenetic Analysis and Target Deletion of *CsPMK1*

The phylogenetic analysis revealed that MAPKs from *C. scovillei*, *C. gloeosporioides*, *S. cerevisiae*, *M. grisea*, and *F. graminearum* were divided into four clades (Supplementary Figure S1A). All MAPKs (other than *S. cerevisiae* Smk1p) were predicted to contain a conserved site (IPR003527) at their N-termini (Supplementary Figure S1A). An NCBI BlastP search revealed that CsPMK1 shared 100% identity with *C. gloeosporioides* CgMK1, 98.6% identity with *M. grisea* PMK1, 98.3% identity with *F. graminearum* MAP1, 60.8% identity with *S. cerevisiae* Kss1, and 59.7% identity with *S. cerevisiae* Fus3 (Supplementary Figure S1B). These results suggest that CsPMK1 is an ortholog of Fus3/Kss1 MAPKs in *C. scovillei*. To investigate the functional roles of *CsPMK1* in fungal development and plant infection of *C. scovillei*, we deleted *CsPMK1 via* homology-dependent targeted gene replacement (Supplementary Figure S2A). The deletion mutants were verified by Southern blot and RT-PCR (Supplementary Figures S2B,C). The complemented strain (*Cspmk1c*), generated by transforming genomic copies of *CsPMK1* into protoplasts of *ΔCspmk1*, showed recovery expression of *CsPMK1* by RT-PCR (Supplementary Figure S2C).

### *CsPMK1* Is Involved in Stress Response of Mycelia

**Figure 1:**
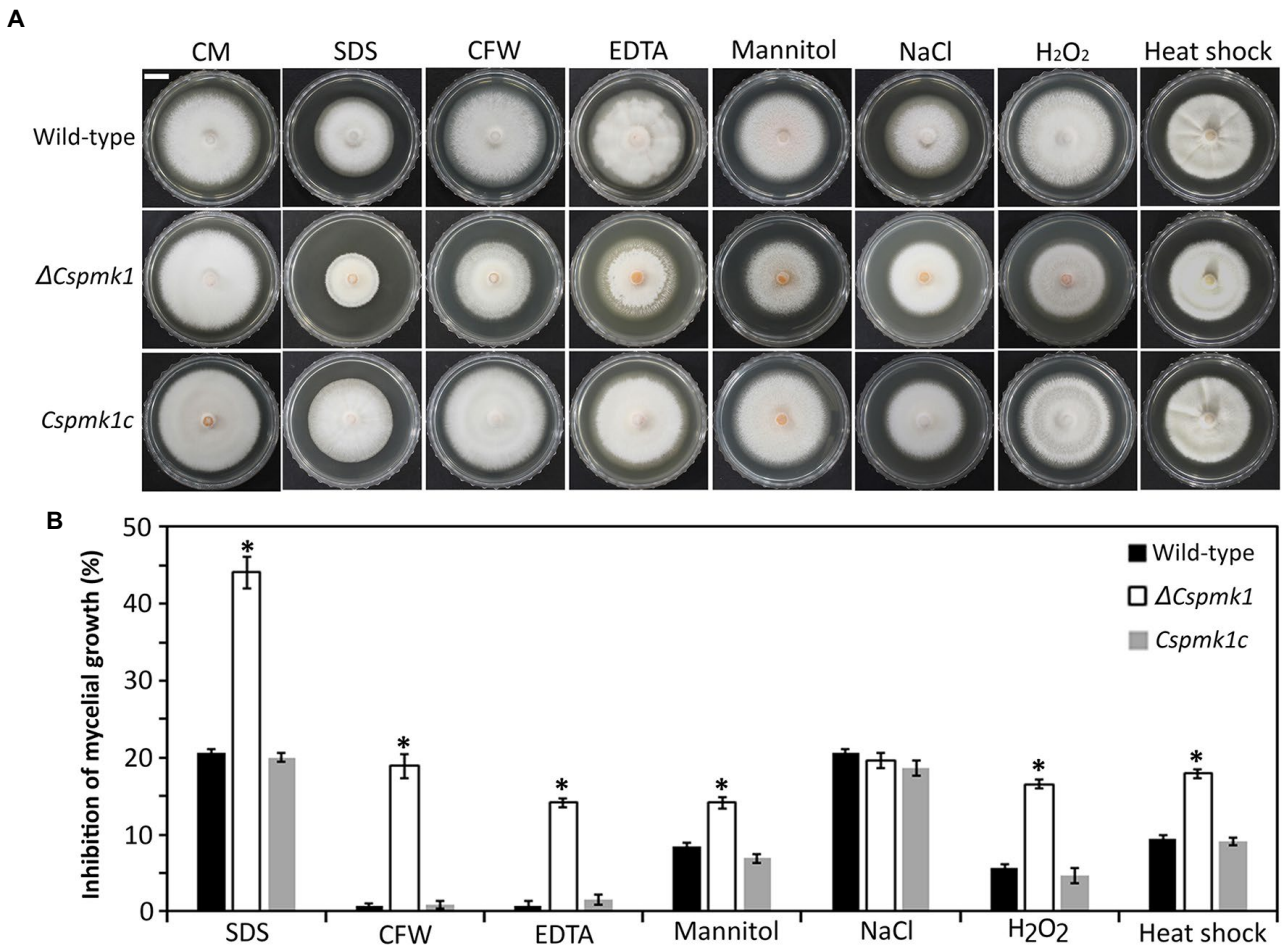
*CsPMK1* is involved in stress adaptation. **(A)** Mycelial growth of the indicated strains on CMA amended with chemical and thermal stresses. Three-day-old mycelial agar plugs (5 mm in diameter) were inoculated into complete medium agar (CMA), and cultured at 28°C, and into CMA containing 0.005% sodium dodecyl sulfate (SDS), 3,000 ppm calcofluor white (CFW), 1 mM ethylenediaminetetraacetic acid (EDTA), 1 M mannitol, 0.4 M NaCl, and 5 mM H_2_O_2_ and cultured at 25°C. Photographs were taken after 6 days. Scale bar, 1 cm. **(B)** Inhibition of mycelial growth under chemical and thermal stresses. Inhibition of mycelial growth was calculated based on colony diameter of on CMA and CMA amended with stresses. Significant differences in a group indicated with esthetic star (*) were estimated using Duncan’s test (*p* < 0.05).

Mycelial growth of *ΔCspmk1* was found to be normal, compared to that of the wild type and *Cspmk1c* (Figure 1). Because orthologs of *CsPMK1* were reported to be involved in stress adaptation of mycelia in *C. fructicola*, *C. gloeosporioides*, and *C. higginsianum* (Wei et al., 2016; He et al., 2017; Liang et al., 2019), we evaluated mycelial growth of *ΔCspmk1* under various stress conditions (Figure 1). The result showed that mycelial growth of *ΔCspmk1* was significantly inhibited by cell wall [0.005% sodium dodecyl sulfate (SDS), 3,000 ppm calcofluor white (CFW), and 1 mM ethylenediaminetetraacetic acid (EDTA)], osmotic (1 M mannitol), oxidative (5 mM H_2_O_2_), and thermal (28°C) stresses, compared to that of the wild type and *Cspmk1c*. Therefore, *ΔCspmk1* was more sensitive to chemical and thermal stresses, implicating that *CsPMK1* may be involved in tolerance to cell wall, osmotic oxidative, and thermal stresses.

### *CsPMK1* Is Important for Conidium Morphology

**Figure 2:**
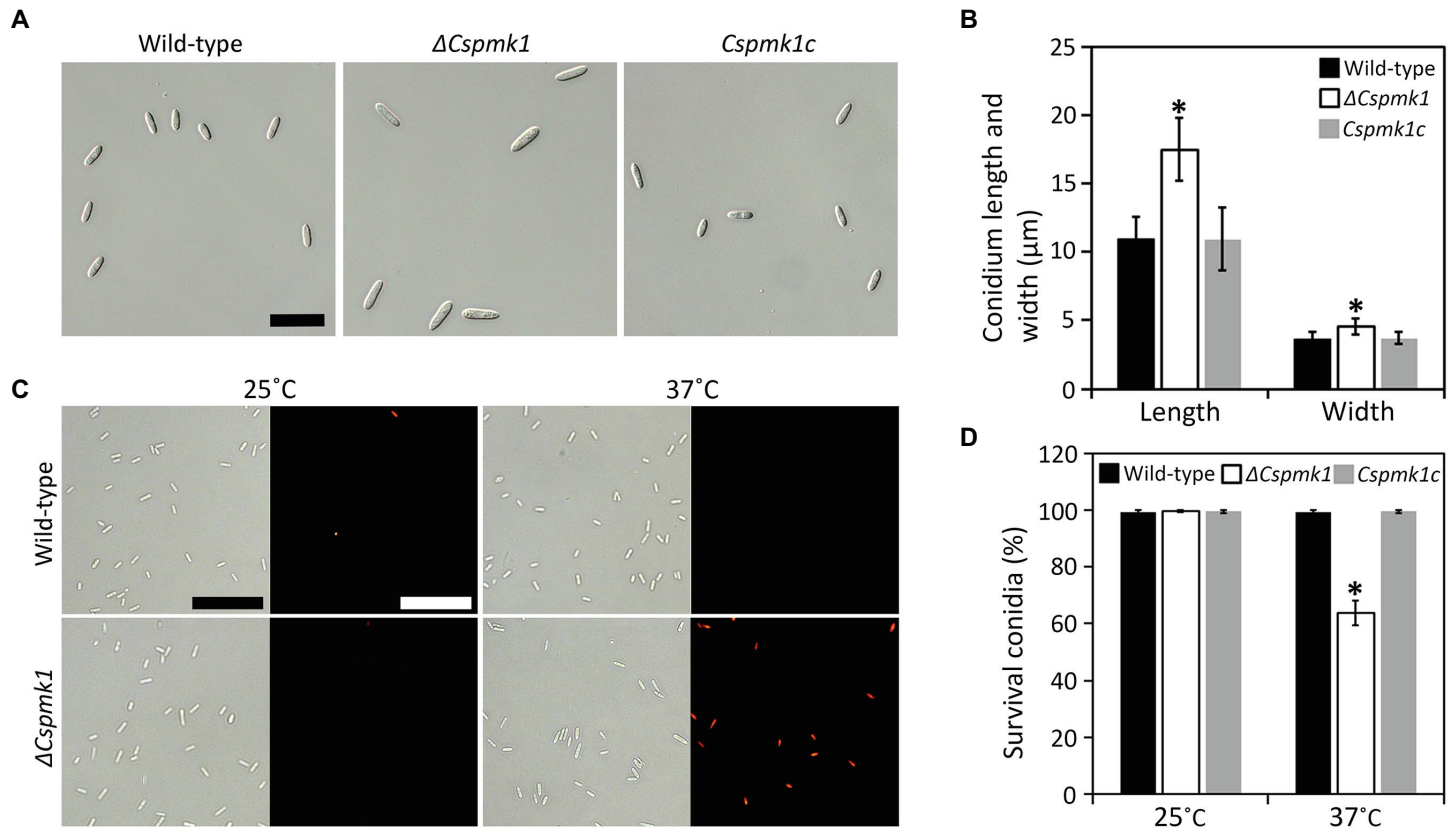
*CsPMK1* is important for conidium morphology and conidium viability under heat shock. **(A,B)** Morphology of conidium. **(A)** Photographs of conidium. Conidia were harvested from 7-day-old oatmeal agar (OMA). Scale bar, 25 μm. **(B)** Quantitative analysis of conidium size. Length and width were measured at least 100 conidia, harvested from 7-day-old OMA. **(C,D)** Conidium viability under heat shock. **(C)** Photographs of conidia stained with phloxine B. Conidia were incubated at 25°C or 37°C for 16 h and then stained with phloxine B (10 μg ml^−1^) for 30 min. Dead conidia showed red fluorescence through fluorescent microscope. Scale bar, 50 μm. **(D)** Quantitative analysis of conidium viability. Dead conidia stained with red color by phloxine B were counted in total of 100 conidia. Significant differences in a group indicated with esthetic star (*) were estimated using Duncan’s test (*p* < 0.05).

Although deletion of *CsPMK1* did not affect conidiation, *ΔCspmk1* was found to produce morphologically abnormal conidia (length: 16.1 ± 2.3 μm; width: 4.5 ± 0.6 μm), which were significantly larger than those of the wild type (length: 11.0 ± 1.6 μm; width: 3.6 ± 0.6 μm) and *Cspmk1c* (length: 11.0 ± 2.3 μm; width: 3.7 ± 0.5 μm; Figures 2A,B). This finding suggests that *CsPMK1* is important for conidium morphology. Because the ortholog of *CsPMK1* was reported to be involved in the conidium life span of *A. nidulans* (Kang et al., 2013), we assayed conidium survival with an application of phloxine B staining. Unexpectedly, conidium viability of *ΔCspmk1* did not differ from that of the wild type and *Cspmk1c* (Figures 2C,D). Next, we assayed conidium survival of *ΔCspmk1* under thermal stress. After incubation at 37°C for 16 h, the conidium survival rate of the wild type and *Cspmk1c* was 99.5 and 99.7%, respectively (Figures 2C,D). However, only 63.8% of conidia of *ΔCspmk1* survived under the same conditions, indicating that *CsPMK1* is related to conidium viability under heat shock. These results suggest that *CsPMK1* is important for *C. scovillei* conidium morphology and conidium survival under heat shock.

### *CsPMK1* Is Essential for Conidial Germination and Appressorium Formation

**Figure 3:**
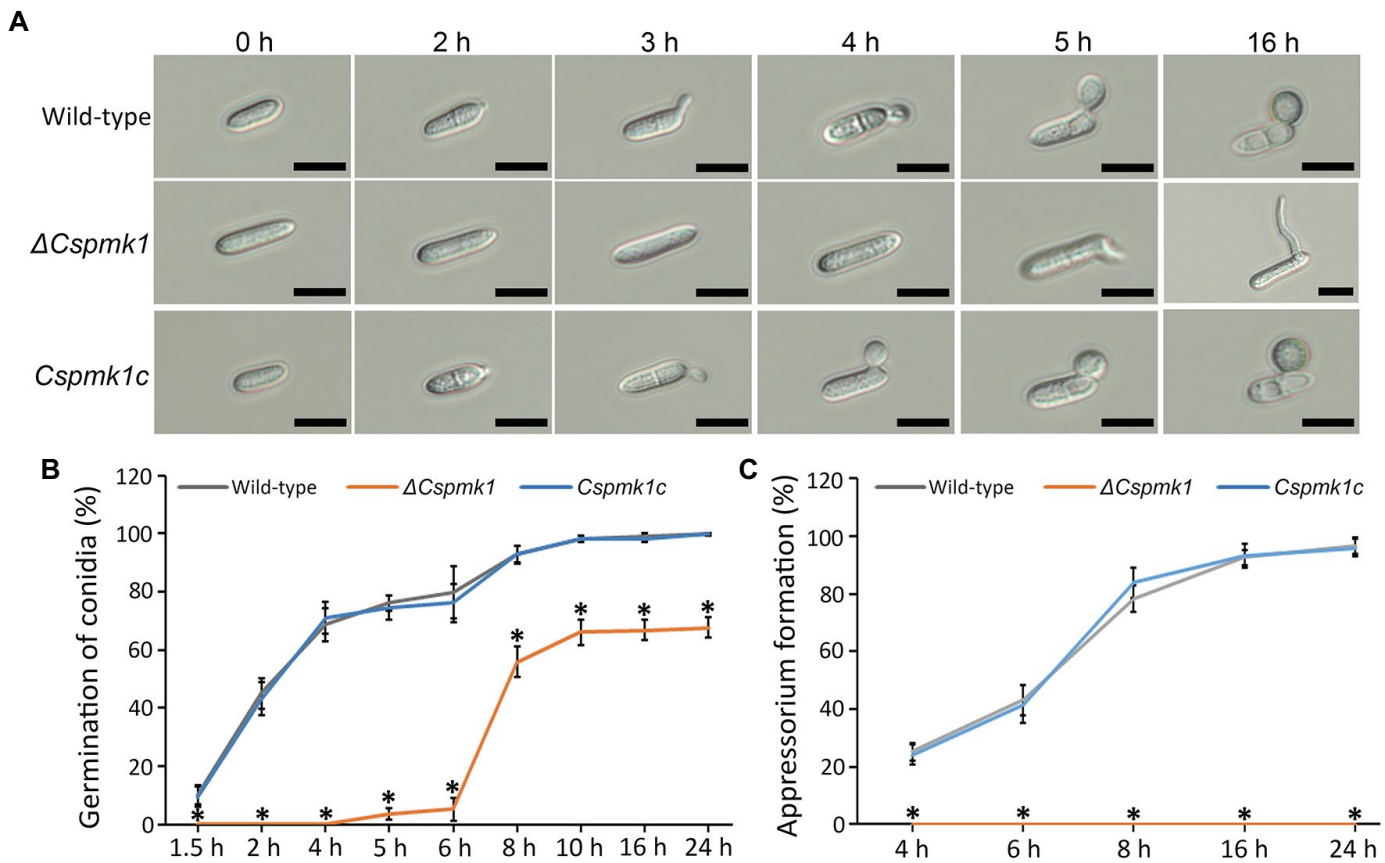
*CsPMK1* is essential for conidial germination and appressorium formation. **(A)** Photographs of conidial germination and appressorium formation. Conidial suspensions (5 × 10^4^ ml^−1^) harvested from 7-day-old oatmeal agar (OMA) were dropped into the hydrophobic surface of coverslips and incubated at 25°C. Scale bar, 10 μm. **(B,C)** Quantitative measurement of conidial germination **(B)** and appressorium formation **(C)**. Conidial suspensions were placed on the hydrophobic surface of coverslips and incubated at 25°C. Significant differences in a group indicated with esthetic star (*) were estimated using Duncan’s test (*p* < 0.05).

To investigate the role of *CsPMK1* in pre-infection development, we evaluated conidial germination and appressorium formation on the hydrophobic surface of coverslips in a time-course manner. The result showed that conidia of the wild type and *Cspmk1c* started to germinate within 2 h. The germination rates of wild-type and *Cspmk1c* conidia increased with prolonging incubation, reaching 98.0 ± 1.0% and 98.0 ± 1.0%, respectively (Figures 3A,B). *ΔCspmk1* conidia started to germinate at 5 h, and the germination rate was 66.1 ± 4.4% at 10 h (Figures 3A,B). Furthermore, prolonged incubation failed to restore conidial germination of *ΔCspmk1*, suggesting that *CsPMK1* is involved in conidial germination, in response to hydrophobic surfaces. After 16 h of incubation on the hydrophobic surface of coverslips, 92.3 ± 3.1% of wild-type and *Cspmk1c* conidia were found to form melanized appressoria. However, the germinated conidia of *ΔCspmk1* failed to differentiate appressoria at 16 h (Figures 3A,C), suggesting that *CsPMK1* is essential for appressorium formation from germ tube. Next, we evaluated whether signaling molecules could recover appressorium formation of *ΔCspmk1*. Exogenous cAMP, CaCl2, and cutin monomers failed to induce swelling and restore appressorium at the germ tube of *ΔCspmk1* strain (Supplementary Table S2). To assess whether appressorium-like structure (ALS) formation from mycelium is affected in *ΔCspmk1*, we placed young mycelia on the hydrophobic surface of coverslips. The result showed that *ΔCspmk1* failed to generate ALS, whereas the wild type and *CsPMK1c* formed many ALSs (Supplementary Figure S3A), suggesting that *CsPMK1* is required for ALS formation of *C. scovillei*. These results suggest that *CsPMK1* is involved in conidial germination and essential for appressorium formation from germ tube and ALS from mycelium.

### *CsPMK1* Is Related to Nuclear Division and Lipid Mobilization

**Figure 4:**
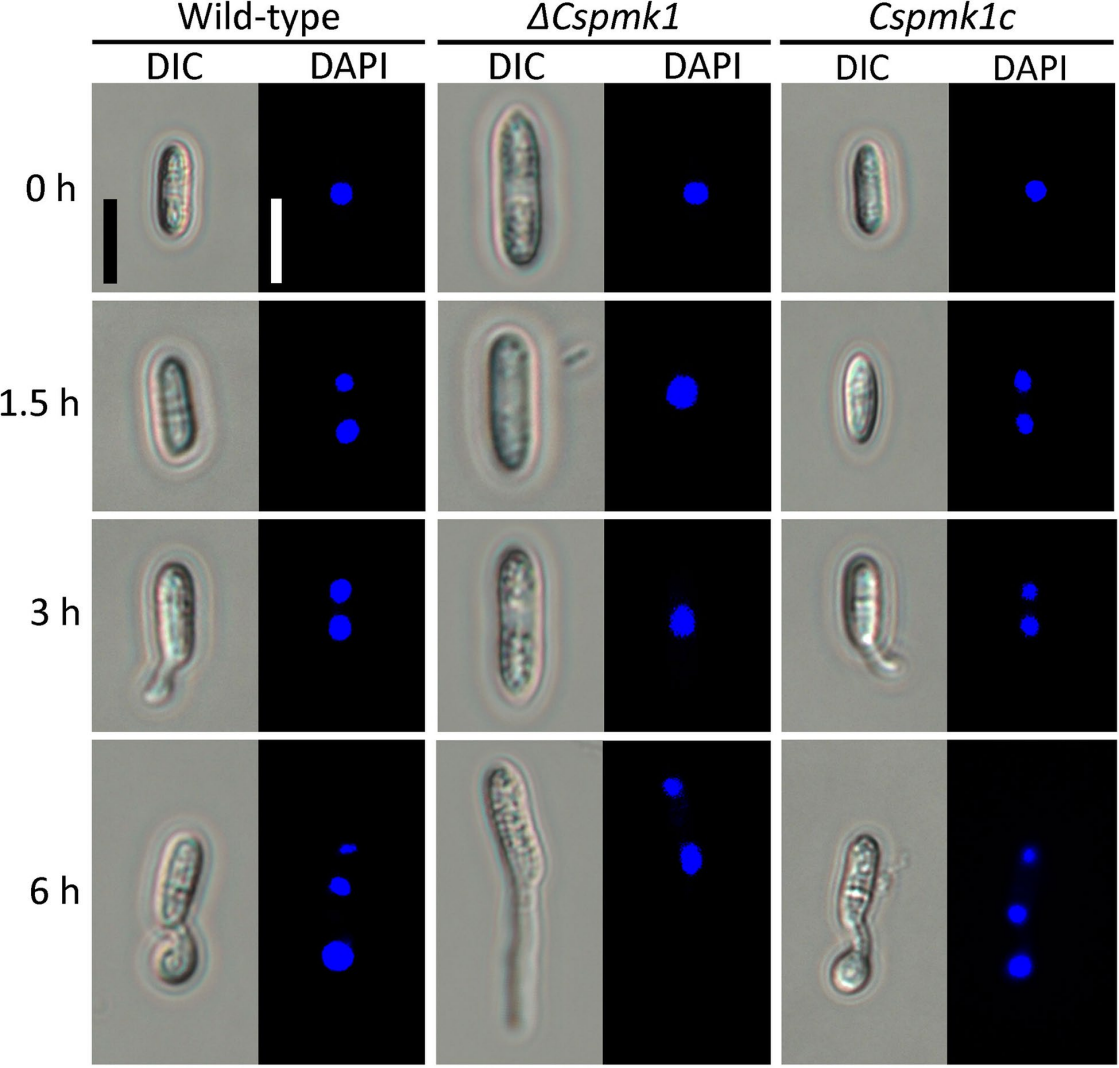
*CsPMK1* is involved in nuclear division. Conidial suspensions (5 × 10^4^ ml^−1^) were placed on the hydrophobic surface of coverslips and incubated at 25°C. The 4′,6-Diamidino-2-phenylindole (DAPI) was used to stain nuclei in developing conidia. Scale bar, 10 μm.

In many fungal species, conidial germination and appressorium formation are tightly coordinated with punctual nuclear division (Serna and Stadler, 1978; Harris, 1999; Saunders et al., 2010). Therefore, we observed nuclear division during appressorium formation on the hydrophobic surface of coverslips. Wild-type conidia showed two nuclei at 1.5 h and three nuclei at 6 h (Figure 4). However, in *ΔCspmk1*, the conidia exhibited two nuclei at 6 h (Figure 4). The delayed nuclear division was restored in *CsPMK1c*. This result suggests that the *CsPMK1* is involved in nuclear division.

**Figure 5:**
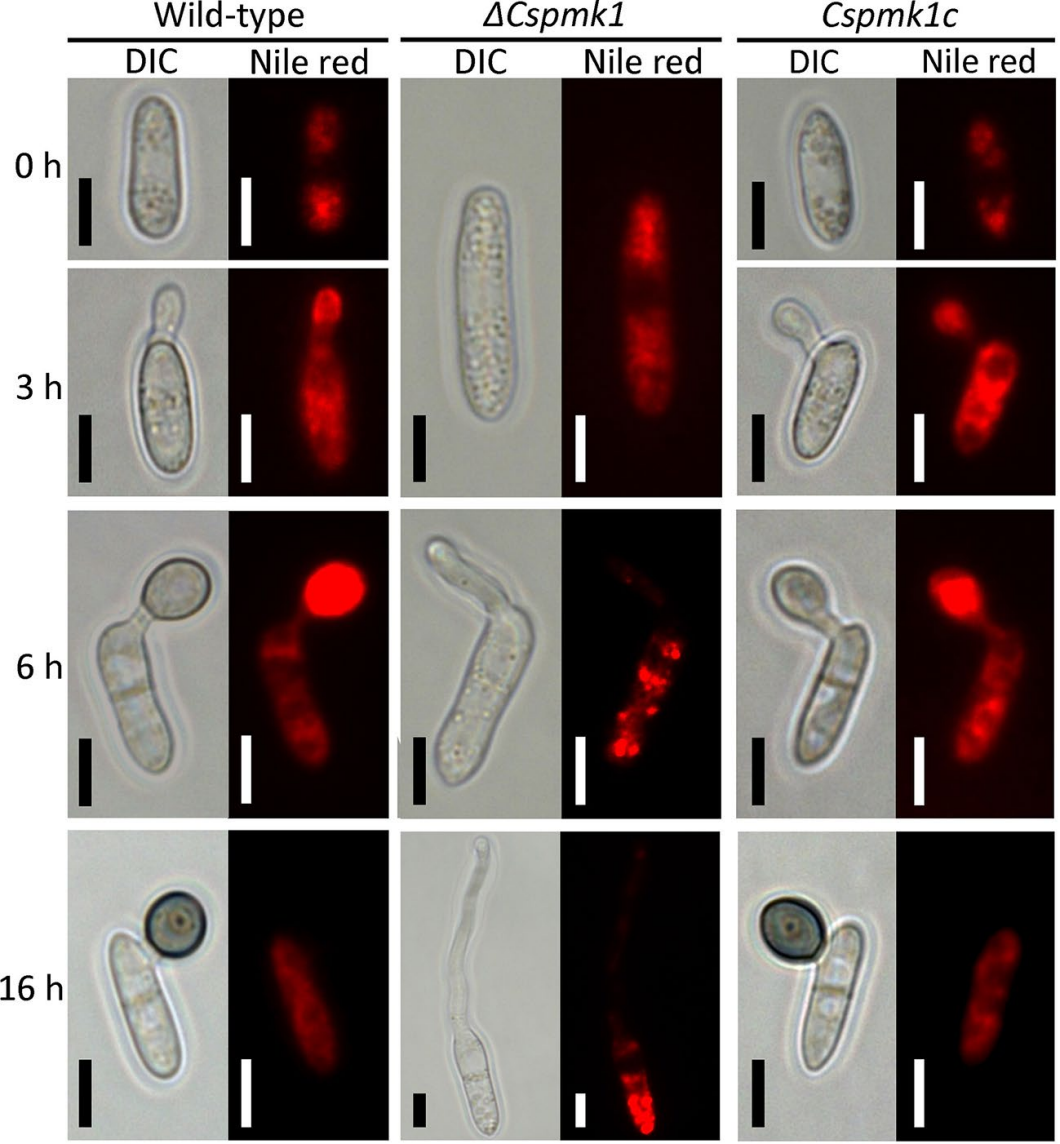
*CsPMK1* is related to lipid mobilization. Conidial suspensions (5 × 10^4^ ml^−1^) were placed on the hydrophobic surface of coverslips and incubated at 25°C. Nile red was used to stain lipid droplets during conidial germination and appressorium formation. Scale bar, 5 μm.

*PMK1* gene was demonstrated to regulate lipid mobilization during appressorium formation of *M. oryzae* (Thines et al., 2000). Therefore, we observed lipid localization during appressorium formation on the hydrophobic surface of coverslips (Figure 5). The result showed that lipid droplets were present in the conidia of *ΔCspmk1*, similar to that in the conidia of the wild type and *Cspmk1c*, suggesting that *CsPMK1* is not involved in lipid storage in conidia. However, lipids failed to translocate into the conidial germ tube and abundantly present in conidia of *ΔCspmk1* at 6 and at 16 h. In contrast, lipids were transported to the germ tube at 3 h and to appressorium at 6 h in the wild type and *Cspmk1c*. Lipids were completely degraded in the appressoria but some remained in conidia of the wild type and *Cspmk1c* at 16 h. Lipids were transported to hyphal tips in *ΔCspmk1*, similar to lipid localization during ALS formation in the wild-type and *Cspmk1c* strains (Supplementary Figure S3B). Therefore, *CsPMK1* plays important roles for nuclear division and lipid mobility during appressorium formation of *C. scovillei*.

### *CsPMK1* Is Critical for Anthracnose Development in Pepper Fruit

**Figure 6:**
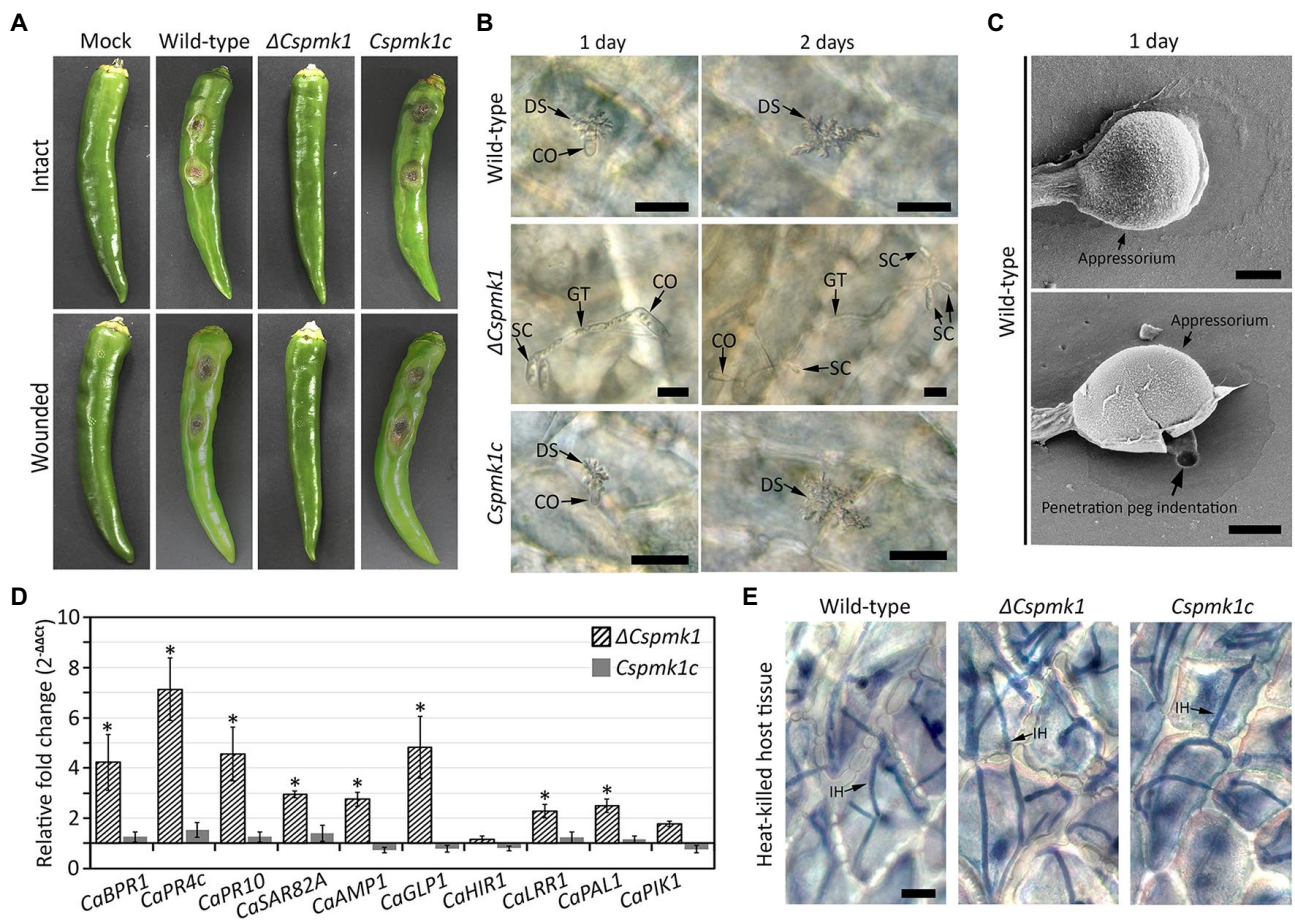
**(A)** *CsPMK1* is critical for pathogenicity. Conidial suspensions (10^6^ ml^−1^) were inoculated into intact and wounded healthy pepper fruits, and incubated in humid plastic boxes at 25°C. Photographs of infected intact and wounded pepper fruits were taken after 9 and 6 days, respectively. **(B)** *CsPMK1* is critical for appressorium formation on plant surface. Conidial suspensions (5 × 10^4^ ml^−1^) were inoculated into intact pepper fruits and incubated in a humid plastic box at 25°C. Host plants infected by wild type and *Cspmk1c* exhibited dendroid structures in the cuticle layer. However, the *ΔCspmk1* failed to develop appressorium. Scale bar, 20 μm. **(C)** Scanning electron microscope (SEM) images of wild-type appressorium and associated biological materials on pepper fruit surface after 1 day. The upper and lower panel shows treatment with 5 and 10 min sonication. Scale bar, 2 μm. **(D)** *CsPMK1* is involved in the suppression of expression of host-defense-related genes (Supplementary Table S3). Wounded pepper fruits were inoculated with conidial suspensions (25 × 10^4^ ml^−1^) and incubated at 25°C for 36 h. The expression of host-defense-related genes was evaluated in pepper fruit tissues infected with *ΔCspmk1*, compared to tissues infected with the wild type and *Cspmk1c*. The pepper actin gene was used as a reference in the qRT-PCR. Significant differences (*) were estimated using Duncan’s test (*p* < 0.05). **(E)** *CsPMK1* is dispensable for invasive growth in immunity-compromised host. Pepper fruits were heated in a drying oven at 65°C for 1 h to obtain heat-killed host tissues. Conidial suspensions (5 × 10^4^ ml^−1^) were delivered into epidermal cells of pepper fruits by syringe and incubated at 25°C for 2 days. Invasive hyphae were stained with blue color using a modified trypan blue solution. Scale bar, 20 μm. CO, SC, GT, DS, and IH indicate conidium, secondary conidium, germ tube, dendroid structure, and invasive hyphae, respectively.

To investigate the role of *CsPMK1* in anthracnose development, we inoculated conidial suspensions into wounded and intact pepper fruits. Both the wild type and *CsPMK1c* induced typical anthracnose disease with sunken lesions on wounded and intact pepper fruits after 6 and 9 days, respectively (Figure 6A). However, *ΔCspmk1* failed to cause typical anthracnose symptoms on both wounded and intact pepper fruits (Figure 6A), suggesting that *CsPMK1* is required for pathogenicity of *C. scovillei*. We further investigated the appressorium-mediated penetration using light microscope and SEM. The result showed that more than 95% of conidia of the wild type and *Cspmk1c* successfully penetrated the host surface within 1 day, whereas *ΔCspmk1* developed secondary conidia instead of appressoria at tip of germ tubes (Figures 6B,C). This finding suggests that *CsPMK1* is essential for appressorium formation in response to a host surface.

The failure of disease formation on wounded pepper fruits revealed that *ΔCspmk1* abolished infectious hyphae growth, possibly resulted from defect in suppression of host immunity. To test this hypothesis, we evaluated host-defense-related genes in the host tissues infected by conidia of *ΔCspmk1*. The host-defense-related genes (*CaBPR1*, *CaPR4c*, *CaPR10*, *CaSAR82A*, *CaAMP1*, *CaGLP1*, *CaLRR1*, and *CaPAL1*) were significantly upregulated in pepper fruits infected by *ΔCspmk1*, compared to that by the wild type and *CsPMK1c* (Figure 6D and Supplementary Table S3). This finding implicates that *CsPMK1* may be involved in the suppression of host-defense mechanisms. To support this hypothesis, we delivered conidial suspensions into epidermal cells of heat-killed pepper fruits by syringe. After 36 h of incubation, *ΔCspmk1* developed a mass of invasive hyphae in the heat-killed host tissues, similar to the wild type and *Cspmk1c* (Figure 6E). This result suggested that the failure of infectious growth by *ΔCspmk1* on wounded pepper fruits was caused by the defect of suppression of host-defense mechanism. These findings suggest that *CsPMK1* is required for anthracnose development by regulating appressorium formation and suppression of host-defense mechanism.

### CsPMK1:sGFP Was Localized to Conidia, Germinating Conidia, and Appressoria

**Figure 7:**
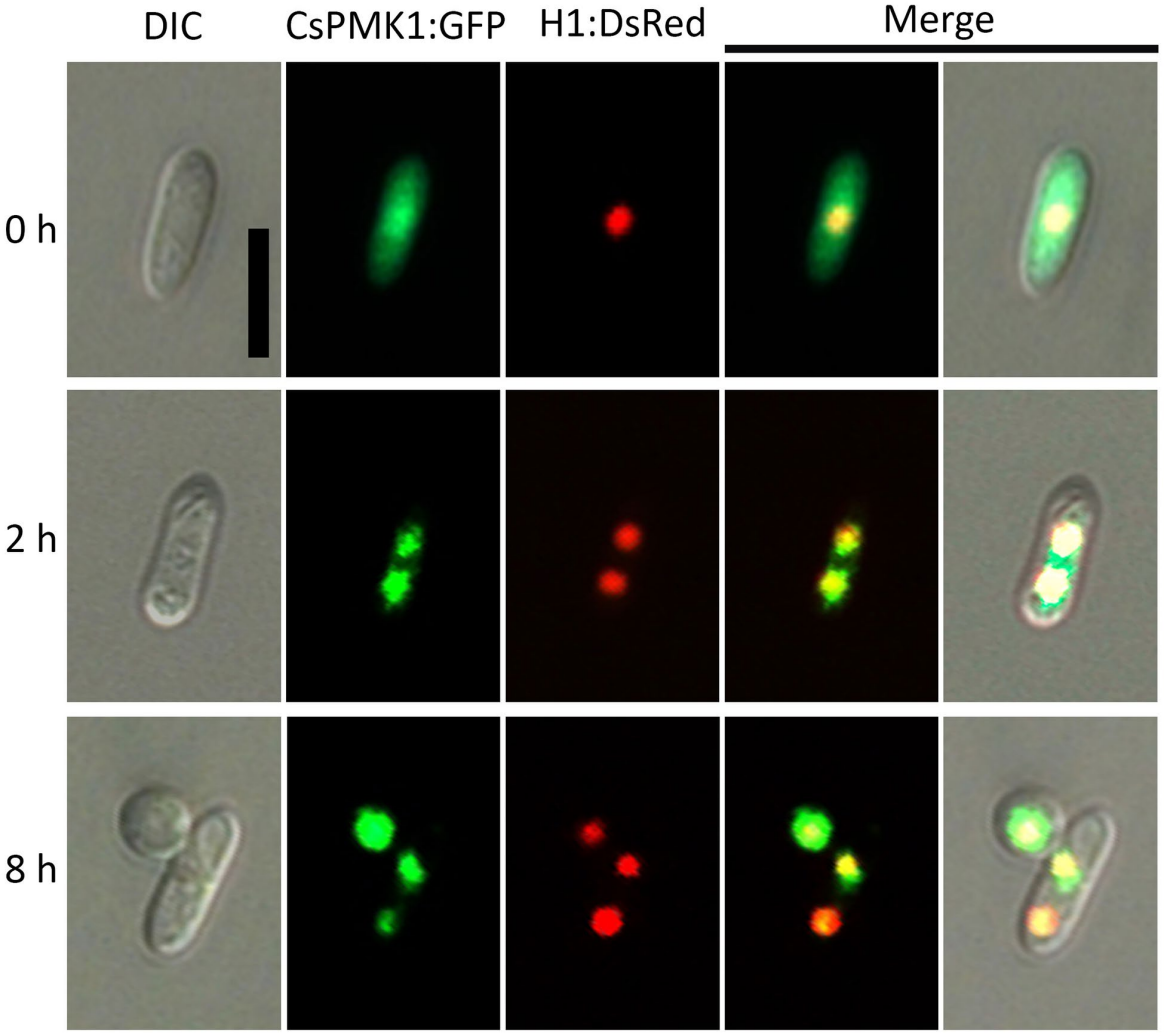
Expression and localization of CsPMK1:GFP fusion protein in *Colletotrichum scovillei*. Conidia obtained from a transformant expressing H1:RFP and CsPMK1:GFP were placed on the hydrophobic surface of coverslips and incubated at 25°C. Scale bar, 10 μm.

To investigate the expression and localization of *CsPMK1* during fungal development, we localized the CsPMK1 in the transformants expressing H1:DsRed fusion protein. The transformants expressing CsPMK1:sGFP exhibited normal fungal development and pathogenicity, compared to the wild type. CsPMK1:sGFP was detected at all life stages of *C. scovillei*. However, a stronger signal was only found in conidia, germinating conidia, and developing appressoria (Figure 7). At 0 h, the CsPMK1:sGFP was detected in the conidia. At 2 h, the CsPMK1:sGFP was found to accumulate in nuclei of conidium. At 8 h, the CsPMK1:sGFP was mainly localized to appressoria but was still detectable in nuclei of conidia (Figure 7).

### CsHOX7 May Be a Putative Target of CsPMK1

**Figure 8:**
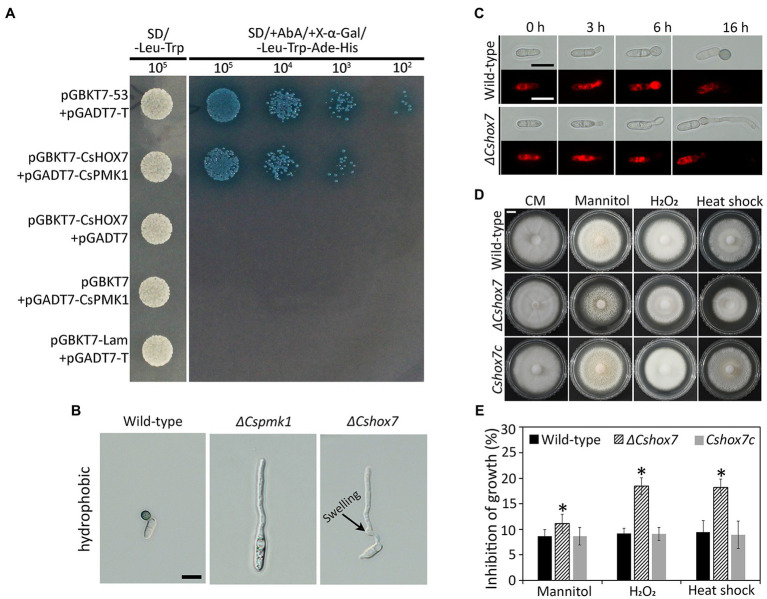
Yeast two-hybrid analysis, appressorium formation assays, lipid mobility, and stress adaption of mycelia. **(A)** Yeast two-hybrid assay. The control strain expressing pGBKT7-53 and pGADT7-T (control), and a strain expressing pGBKT7-CsHOX7 and pGADT7-CsPMK1 was able to grow on both SD-Leu-Trp and SD/ +Aureobasidin A/+ X-α-Gal/ −/−Leu-Trp-Ade-His. A strain expressing pGBKT7 and pGADT7-CsPMK1, pGBKT7-CsHOX7 and pGADT7, or pGBKT7-Lam and pGADT7-T only grew on SD-Leu-Trp. **(B)** Appressorium formation assay. Conidia harvested from 7-day-old oatmeal agar (OMA) were placed on the hydrophobic surface of coverslips and incubated at 25°C. Both *ΔCspmk1* and *ΔCshox7* failed to form appressoria, but *ΔCshox7* developed swellings in germ tube. Scale bar, 10 μm. **(C)** *CsHOX7* is related to lipid mobilization during appressorium formation. Conidial suspensions (5 × 10^4^ ml^−1^) were placed on the hydrophobic surface of coverslips and incubated at 25°C. Nile red was used to stain lipid droplets during conidial germination and appressorium formation. Scale bar, 10 μm. **(D,E)** Mycelial growth of *ΔCshox7* on complete medium agar (CMA) containing chemical stresses. Three-day-old mycelial agar plugs (5 mm in diameter) were inoculated into complete medium agar (CMA) and cultured at 28°C, or on CMA containing 1 M mannitol and 5 mM H_2_O_2_. **(D)** Photographs were taken after 6 days. Scale bar, 1 cm. **(E)** Inhibition of mycelial growth was calculated based on colony diameter on CMA and CMA amended with stresses. Significant difference (*) was estimated using Duncan’s test (*p* < 0.05).

Homeobox transcription factor (HOX7) was previously reported as a main regulator of appressorium formation in several fungal species (Kim et al., 2009; Yokoyama et al., 2018; Fu et al., 2021). Therefore, we evaluated expression levels of *CsHOX7* in *ΔCspmk1* and wild-type strains. The *CsHOX7* was found to be not significantly expressed in the *ΔCspmk1*, compared to wild type, during appressorium formation on the hydrophobic surface of coverslips (Supplementary Figure S4A). This result suggests that CsPMK1 may not regulate expression of *CsHOX7* during appressorium formation of *C. scovillei*. We further tested the interaction between CsPMK1 and CsHOX7 by using a yeast two-hybridization (Y2H) analysis. The result showed that transformants containing the recombinant prey vector (pGADT7-CsPMK1) and bait vector (pGBKT7-CsHOX7) grew on double dropout agar (SD-Leu-Trp) and quadruple dropout agar (SD-Leu-Trp-His-Ade/X-α-Gal/Aureobasidin A), as did transformants containing positive control vectors (Figure 8A). However, transformants containing negative control vectors failed to grow on quadruple dropout agar (Figure 8A). These results suggest that *CsPMK1* may interact with CsHOX7. To test this hypothesis, we predicted the putative phosphorylation sites in the amino acid sequence of CsHOX7 using NetPhos 3.1. The result revealed that five putative sites in CsHOX7 may be phosphorylated by MAPK (Supplementary Figure S4B). Furthermore, we compared appressorium formation of *ΔCspmk1* and a *CsHOX7* deletion mutant (*ΔCshox7*) and investigated the lipid metabolism and stress adaptation of *ΔCshox7*. The result showed that both *ΔCspmk1* and *ΔCshox7* failed to form appressorium. Unlike *ΔCspmk1*, *ΔCshox7* developed swelling on the germ tube (Figure 8B), indicating that CspMK1 and CsHOX7 play different roles in recognition of surface signals. The lipids staining showed that lipids were not transported into the germ tube of *ΔCshox7* during appressorium development (Figure 8C), suggesting that *CsHOX7* is important for lipid mobility. The mycelial growth of *ΔCshox7* was significantly inhibited by osmotic (mannitol), oxidative (H_2_O_2_), and thermal (28°C) stresses, compared to that of the wild type and *Cshox7c* (Figures 8D,E), suggesting that CsHOX7 plays an important role in stress adaption of mycelia. Together, these results indicate that CsHOX7 may be a putative target of CsPMK1 in *C. scovillei*.

## Discussion

Although the PMK1-type MAPK has been investigated in more than 20 fungal species (Jiang et al., 2018), its role in anthracnose development in the *C. scovillei*-pepper fruit pathosystem is unclear. Therefore, we set out to investigate the functional role of *CsPMK1* in the development and pathogenicity of *C. scovillei*. The results revealed that *CsPMK1* plays essential roles in appressorium formation and plant infection. *CsPMK1* is important for morphology and germination of conidium, and tolerance to stress conditions. Moreover, functions of *CsPMK1* are associated with lipid metabolism and nuclear division, which are essential to develop functional appressorium. Our study implicated pleiotropic roles of *CsPMK1* in fungal developments and pathogenicity in the *C. scovillei*-pepper fruit pathosystem.

In many plant pathogenic fungi, the Slt2 and Hog1-MAPKs are known to regulate stress responses (Jiang et al., 2018). However, increasing evidence has revealed that the Fus3/Kss1-MAPK coordinates with Slt2- and Hog1-related MAPKs to control stress adaption (Segorbe et al., 2017). For example, deletion of *ChMK1* in *C. higginsianum*, orthologous to *Fus3/Kss1*, increases sensitivity to cell wall stresses (Wei et al., 2016). In *C. gloeosporioides* and *Corynespora cassiicola*, orthologs of PMK1 were reported to be associated with tolerance to osmotic stress (He et al., 2017; Liu et al., 2017). Consistently, *ΔCspmk1* was found to be hypersensitive to multiple stresses, including cell wall integrity stress (SDS, CFW, and EDTA), osmotic stress (mannitol), oxidative stress (H_2_O_2_), and thermal stress (28°C; Figure 1). Therefore, *CsPMK1* may cooperate with other signaling pathways to regulate the stress response in *C. scovillei*.

Deletion of *CsPMK1* caused defects in morphology and germination of conidium in *C. scovillei*. Conidial germination was found to be delayed and reduced in *ΔCspmk1* (Figures 3A,B), implicating that *CsPMK1* is involved in regulation of conidial germination. Similar results have been reported previously: deletion of *PMK1* orthologs in *C. fructicola* and *C. lagenarium* caused significant reduction in conidial germination (Takano et al., 2000; Liang et al., 2019). However, the PMK1 orthologs in *C. gloeosporioides* and *C. truncatum* are not involved in conidial germination (Xiong et al., 2015; He et al., 2017). This phenotypic divergence reveals various roles of *PMK1* in conidial germination among *Colletotrichum* species. Interestingly, the *ΔCspmk1* produced significantly larger conidia, than the wild type and *CsPMK1c* (Figures 2A,B), which represents a novel function of *PMK1* in fungi. In *A. nidulans*, *MpkB*, orthologous to *PMK1*, was reported to be involved in spore viability, which was indicative to test conidium longevity of *ΔCspmk1*. Unexpectedly, conidium survival was not altered in *ΔCspmk1*, compared to the wild type (Figures 2C,D). However, treatment of heat shock significantly reduced conidium survival rate in *ΔCspmk1*, compared to that of the wild type and *Cspmk1c* (Figures 2C,D), suggesting that *CsPMK1* may be involved in conidium viability under heat shock.

Deletion of *CsPMK1* abolished appressorium formation of *C. scovillei* (Figure 3), which is consistent with reports involving other plant pathogenic fungi (Xu and Hamer, 1996; Takano et al., 2000; Ruiz-Roldán et al., 2001; Xiong et al., 2015; Wei et al., 2016; He et al., 2017). The defect in appressorium formation of *ΔCspmk1* was not restored by exogenous additions of cAMP, CaCl_2_, and cutin monomers (Supplementary Table S2). To differentiate an appressorium, the wild-type conidium undergoes two rounds of mitosis. Notably, the nuclear division in *ΔCspmk1* conidia was delayed during conidial germination and appressorium formation (Figure 4). However, successive rounds of nuclear division occurred in the germ tube of *ΔCspmk1*, suggesting that *CsPMK1* may be involved in regulating of mitosis at the early stages of conidial germination and appressorium formation. Lipids are essential to build up turgor pressure in appressorium maturation (Schadeck et al., 1998; Thines et al., 2000). Lipid mobility was found to be defective during both appressorium formation and ALS formation of *ΔCspmk1* (Figure 5), suggesting that *CsPMK1* may play important role in lipid mobilization and mobility of lipid during appressorium formation. Transcription factors, MST12 and MoSFL1, were previously demonstrated to be direct downstream targets of PMK1 (Park et al., 2002; Li et al., 2011). Interestingly, deletion of either *MST12* or *MoSFL1* did not affect appressorium formation (Park et al., 2002; Li et al., 2011), indicating that *PMK1* regulates appressorium formation *via* other downstream components. Orthologs of *CsHOX7* were reported to regulate appressorium formation in several plant pathogenic fungi (Kim et al., 2009; Yokoyama et al., 2018; Fu et al., 2021). Recently, Pmk1 was demonstrated to regulate appressorium formation *via* phosphorylation of MoHOX7 in *M. oryzae* (Osés-Ruiz et al., 2021). The CsHOX7 was also found to interact with CsPMK1, based on the result of a Y2H assay (Figure 8A). In addition, we also found that *ΔCshox7* was defective in lipid mobility and tolerance to stress conditions (Figures 8C–E). In response to hydrophobic surface, the *ΔCshox7* failed to form appressorium but developed swellings on germ tube (Fu et al., 2021), which was not observed in *ΔCspmk1* (Figure 8B). Appressorium development of *ΔCshox7* was enhanced with exogenous additions of signal molecules (Fu et al., 2021). These results suggested that CsPMK1 may control appressorium formation through CsHOX7 and regulates surface recognition *via* other transcription factors in *C. scovillei*. *ΔCspmk1* conidia were non-pathogenic to intact pepper fruits, due to abolishment of appressorium formation on host surface (Figures 6A,B). Our study of appressorium-mediated penetration implicated that *C. scovillei* has evolved a different strategy to penetrate host cuticle during anthracnose development (Fu et al., 2021). For many other foliar plant pathogenic fungi, the appressorium peg directly reaches invading host cells using strong mechanical force, which is well studied in rice blast fungus, *M. oryzae* (Howard and Valent, 1996; Soanes et al., 2012). However, *C. scovillei* forms a tiny penetration peg into a thick cuticle layer, which does not reach epidermal cells of pepper fruits (Figures 6B,C). Subsequently, the fungus induces dendroid structures in the cuticle layer and maintains the shape of appressoria. *ΔCspmk1* conidia failed to induce anthracnose on wounded pepper fruit, possibly because of defect in suppression of host immunity. This hypothesis could be supported by the significant upregulation of host-defense-related genes in host tissue infected by *ΔCspmk1* conidia (Figure 6D), in addition to the recovery of invasive hyphae growth of *ΔCspmk1* in the heat-killed host tissues (Figure 6E). Considering that *M. oryzae PMK1* controls the expression of effectors to prevent plasmodesmal closure during invasive hyphae spread in host tissue (Sakulkoo et al., 2018), the PMK1-type MAPKs may play highly conserved roles in suppression of host immunity during diseases.

Taken together, our results demonstrated the regulatory role of *CsPMK1* in fungal stress adaption, morphological development, and suppression of host defenses in the *C. scovillei*-pepper fruit pathosystem. Our findings provide insights into the molecular mechanisms underlying development of fruit anthracnose. Deletion mutants of *CsPMK1* can serve as genetic resources for further study of the *C. scovillei*-pepper fruit pathosystem.

## Data Availability Statement

The raw data supporting the conclusions of this article will be made available by the authors, without undue reservation.

## Author Contributions

TF and KK conceived and designed the study and prepared the manuscript. TF, J-HS, N-HL, and KL performed the experiment and analyzed the data. All authors contributed to the article and approved the submitted version.

## Funding

This study was supported by the Basic Science Research Program through the National Research Foundation of Korea grant (NRF-2020R1A2C100550700) funded by the Ministry of Education, Science and Technology, and by a grant (918019043HD020) from the Strategic Initiative for Microbiomes in Agriculture and Food, Ministry of Agriculture, Food and Rural Affairs, Republic of Korea. TF and N-HL were supported by a graduate fellowship through the Brain Korea 21 Program. The funders had no role in study design, data collection and analysis, decision to publish, or preparation of the manuscript.

## Conflict of Interest

The authors declare that the research was conducted in the absence of any commercial or financial relationships that could be construed as a potential conflict of interest.

## Publisher’s Note

All claims expressed in this article are solely those of the authors and do not necessarily represent those of their affiliated organizations, or those of the publisher, the editors and the reviewers. Any product that may be evaluated in this article, or claim that may be made by its manufacturer, is not guaranteed or endorsed by the publisher.

## Footnotes

^1^ http://cfgp.riceblast.snu.ac.kr/
