# Supplementary material for: Mitogen-Activated Protein Kinase CsPMK1 Is Essential for Pepper Fruit Anthracnose by Colletotrichum scovillei
Source: Front Microbiol. 2022 Feb 24;13:770119. doi: 10.3389/fmicb.2022.770119 (PMC8907736; doi:10.3389/fmicb.2022.770119)
Supplement: Supplementary file 1 [file Data_Sheet_1.pdf]

Supplementary Material

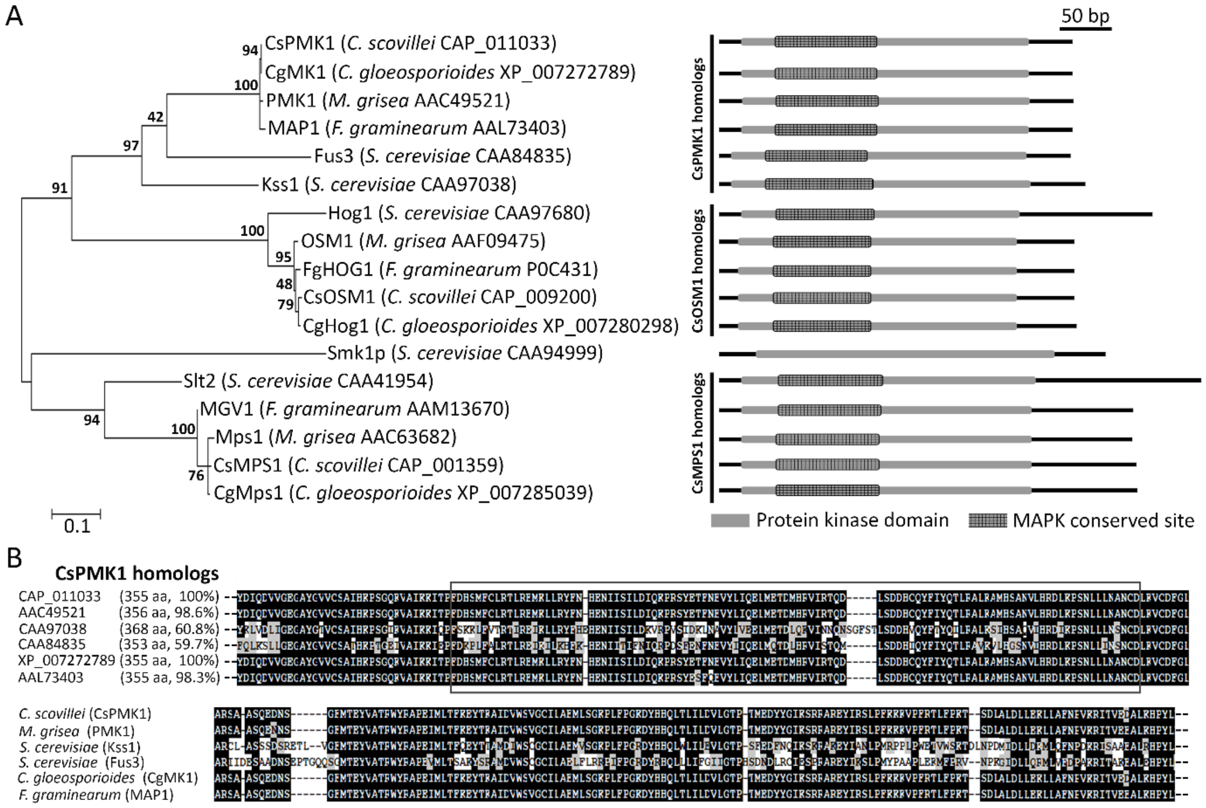

Figure S1. Analysis of phylogenetic relationship, domain representation, and conserved amino acid sequence. (A) Phylogenetic analysis and domain representation of CsPMK1 and its homologs. The phylogenetic tree was constructed by using a maximum-likelihood method with 1000 bootstraps in the MEGA 7 software. Domain structures, including a protein kinase domain (IPR000719) and MAPK conserved site (IPR003527), were predicted using InterProScan. (B) Alignment of conserved amino acid sequences of MAPKs. The identity from NCBI Blastp of each protein follows its name. Black shadow and black box indicate conserved amino acid and MAPK conserved site, respectively.

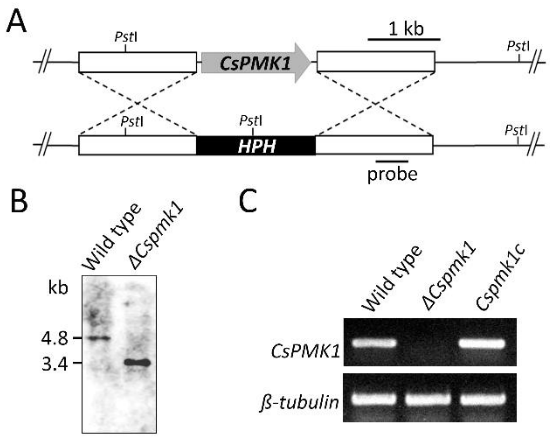

Figure S2. Targeted deletion of *CsPMK1* gene. (A) Targeted deletion of *CsPMK1*. The *CsPMK1* was replaced by the *HPH* cassette, and restriction enzyme *Pst*I was used to digest genomic DNA. (B) Verification of *CsPMK1* deletion by Southern blot. Genomic DNA in the indicated strains was digested with *Pst*I and hybridized to a 500 bp probe. (C) Expression of *CsPMK1* in the targeted deletion mutant. Expression of *CsPMK1* was verified by RT-PCR. Total RNA was extracted from mycelia of the wild-type and transformants.

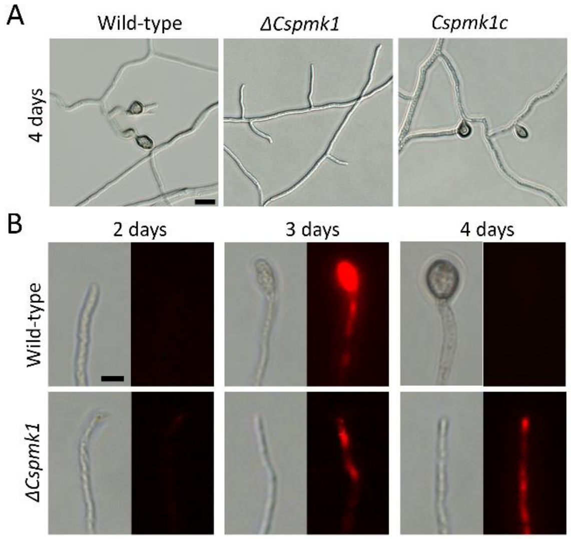

Figure S3. Observation of appressorium-like structure (ALS) formation and lipid mobility during ALS development. (A) Observation of ALS formation. Three-day old oatmeal agar (OMA) containing

mycelia was placed on hydrophobic coverslips, and incubated in a humid plastic box at 25°C without light. Photographs were taken after 4 days. Scale bar, 10 µm. (B) Lipid staining in ALS development. The lipids were stained in hyphal tips and ALS with Nile red at 2, 3, and 4 days. Scale bar, 5 µm.

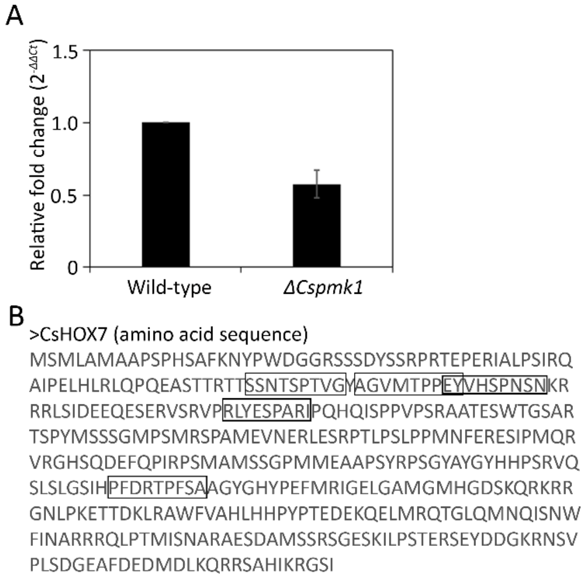

Figure S4. Expression of *CsHOX7* gene and putative phosphorylation sites of CsHOX7 protein. (A) The expression of *CsHOX7* in *ΔCspmk1* and wild-type. Total RNA was extracted from fungal tissues during appressorium development at 8 hpi, in response to the hydrophobic surface of coverslips. The expression levels of *CsHOX7* were measured in a qRT–PCR analysis, and are normalized to *β-tubulin* gene and expressed as relative values with 1 in wild-type. The *CsHOX7* was not significantly expressed in *ΔCspmk1* versus wild-type, because the expression of *CsHOX7* in *ΔCspmk1* was not less than 0.5-fold compared to that in wild-type. (B) Putative phosphorylation sites of mitogen-activated protein kinase (MAPK) in the CsHOX7 protein. Five putative phosphorylation sites (marked with blackbox) were predicted in the amino acid sequence of CsHOX7 as a score of > 0.5 using NetPhos 3.1 (http://www.cbs.dtu.dk/services/NetPhos/).

Table S1. Primers used in this study

Primers	Sequence (5’ → 3’)
**CsPMK1**	
5F	ACCTATCTTGTCCCTCGTTTG
5R	CCTCCACTAGCTCCAGCCAAGCCGATGGTGTGGTCGATGTGGAGA
3F	GTTGGTGTCGATGTCAGCTCCGGAGGAACAGAAGCAAGCGAGAGA
3R	TACGCAGCACAGATACGAAG
NF	CCTCACCTCACCTCACCTCA
NR	GGAGACTGGGACTAGGCGTT
SF	CGATCCCACCCTTCCTTTC
SR	TCTCCTATCCGTCCCGTATG
PF	CCTCTATTCTCTGGATGCTCTG
PR	GCAGGTAATTTGTCGGTTGAG
RTF	TGTGGTCTGTTGGCTGTATTC
RTR	CTTGCTCAGGTTGTCCTTGT
**Hygromycin phosphotransferase**	
HPH_F	GGCTTGGCTGGAGCTAGTGGAGG
HPH_R	CTCCGGAGCTGACATCGACACCAAC
**G418**	
GenF	AGAAGATGATATTGAAGG
GenR	CTCTAAACAAGTGTACCTGTGC
**qRT-PCR**	

β-tubF	AAGCTCGCCGTCAACATGG
β-tubR	CGACGGAACATGGCAGTGAA
CsHOX7_qRTF	GCCGTGTCCCAAGACTTTA
CsHOX7_qRTR	GCTTGTCAGTCGTCTCCTTT
CaActin_F	AAGCTCTCCTTTGTTGCTGTT
CaActin_R	GACTTCTGGGCATCTGAATCT
CaBPR1_F	CAGGATGCAACACTCTGGTGG
CaBPR1_R	ATCAAAGGCCGGTTGGTC
CaPR4c_F	ATGGAGAGTGTTAACAAGTTGTGTGTAG
CaPR4c_R	GCAGTTGACAAATTCATAGTTGACTATAA
CaPR10_F	TGACCTTTGTCGAAGGTGGT
CaPR10_R	GTAAGTAAACTTGTTATATTC
CaSAR82A_F	CAGGGAGATGAATTCTGAGGC
CaSAR82A_R	CATATGAACCTCTATGGATTTCTG
CaAMP1_F	ATGATGAATGCTAATGGATTTAGCGGT
CaAMP1_R	TTAGACCTGATCAATGGGTTCTGTCCTGT
CaGLP1_F	AGTCTTGGTTGCTCTGAGGTCACA
CaGLP1_R	TTAAACCTGTACTTTTATAAATGCG
CaHIR1_F	GACAAAGCTAATGAAGCATTCTAC
CaHIR1_R	GGTGTCGAAGTACTGGGTTACC
CaLRR1_F	GAATGCAACTCCGAAGGG
CaLRR1_R	CTGATAATCTATTACTATTCAATCTCA

CaPAL1_F	GGTTTTGGTGCAACATCACATAGGAG
CaPAL1_R	ATTGTCAAAGTTCTCTTAGCTACTTGGC
CaPIK1_F	GGCTCTTGGTTCACTGGAAGATCATCTA
CaPIK1_R	GCACAGTATCCATATGTACCCATCACTCTG
**CsPMK1:GFP**	
PMK1_F	AACCGCGTTGTTCTCTTCCGGGCC
PMK1_R	CCGCATGATCTCCTGGTAGATCAA
pIG-PMK1_F	CAGGAGATCATGCGGATGGTGAGCAAGGGCGAGGAG
pIG-PMK1_R	GAGAACAACGCGGTTCAACATACGAGCCGGAAGCAT
**Yeast two-hybridization**	
p-PMK1_F	GTGATATGCAGAATTCATGTCGCGCGCGAATCCCCC
p-PMK1_R	TATGGCCATAGAATTCTCACCGCATGATCTCCTGGTAGAT
p-HOX7_F	GTGATATGCAGAATTCATGTCTATGCTCGCCATGGCTGC
p-HOX7_R	TATGGCCATA-GAATTC-CTAAATGCTCCCCCTCTTGATG

Table S2. Effects of signaling molecules on appressorium formation.

Strain	Appressorium formation (%)				
	**dH_2O**	**cAMP**	**$CaCl_2$**	**Cutin monomers**	**Treatment of all**
Wild-type	92.3 ± 3.1	92.8 ± 4.8	92.5 ± 3.4	91.9 ± 4.3	93.1 ± 3.7

ΔCspmk1	0	0	0	0	0
Cspmk1c	90.7 ± 3.1	91.3 ± 3.2	91 ± 2.6	91.5 ± 4.1	92.7 ± 4.1

Effects of exogenous additions of signaling molecules on appressorium formation. Appressorium formation of *ΔCspmk1* was failed to be restored by signaling molecules. Conidial suspension (5×10^4 mL^{-1}) were placed on the hydrophobic surface of coverslips, and mixed with following chemicals with final concentrations (5 mM cAMP, 0.5 mM CaCl2, and 50 µM cutin monomers). Appressorium formation was observed after 24 h.

Table S3. Summary of functions of host defense-related genes in this study

Gene name	Description of functions	Reference
CaBPR1	*CABPR1* is upregulated by infection of *Phytophthora capsica* and *avirulent Xanthomonas campestris pv. vesicatoria*, and treatment of ethylene, salicylic acid (SA), nitric oxide, high salinity, drought stress and low-temperature stress.	1
CaPR4c	*CaPR4c, positively regulating* H_2O_2 accumulation and HR cell death, is upregulated by infection of *avirulent Xanthomonas campestris pv. vesicatoria*.	2
CaPR10	*CaPR10* is upregulated by infection with *avirulent Xanthomonas campestris pv. vesicatoria*. Overexpression of *CaPR10* partially induced HR cell death.	3
CaSAR82A	*CaSAR82A* is upregulated by infection of *Colletotrichum coccodes*, *Phytophthora capsica* and *Xanthomonas campestris pv. vesicatoria*, and treatment of ethylene, salicylic acid, abscisic acid, hydrogen peroxide, methyl jasmonate, indole-3-acetic acid, benzothiadiazole, DL-β-n-amino butyric acid, high salinity, and drought stress and cold stress, but not mechanical wounding.	4
CaAMP1	*CaAMP1* is upregulated by infection of pathogens and exposure to abiotic elicitors. The *CaAMP1* protein shows broad-spectrum antimicrobial activity against bacteria and fungi. *CaAMP1* silencing enhances susceptibility to infection by *Colletotrichum coccodes* and *Xanthomonas campestris pv. vesicatoria*, accompanied by downregulation of *CaBPR1* and *CaPR10.*	5
CaGLP1	*CaGLP1* is upregulated by infection of *Xanthomonas campestris pv. vesicatoria* infection. Silencing of *CaGLP1* enhanced susceptibility to *Xanthomonas campestris pv. vesicatoria*, and caused defection in accumulation of H_2O_2 and induction of cell death during incompatible *Xcv* infection.	6

CaHIR1	*CaHIR1* positively regulates programmed-cell death responses during infection of *Xanthomonas campestris pv. vesicatoria*.	7
CaLRR1	*CaLRR1* is upregulated by treatments of high salinity, abscisic acid and mechanical wounding, but not SA, methyl jasmonate and ethylene. Overexpression of *CaLRR1* enhances *CaPR10* triggered HR cell death, but suppresses *CaHIR1* induced HR cell death.	8
CaPAL1	*CaPAL1* positively regulates SA-dependent defense signaling. Silencing of *CaPAL1* increases susceptibility to *Xanthomonas campestris pv. vesicatoria* infection, and significantly reduced ROS burst, HR cell death, SA accumulation.	9
CaPIK1	*CaPIK1* is upregulated by infection of *Xanthomonas campestris pv. vesicatoria*. Silencing of *CaPIK1* attenuates salicylic acid-dependent defense response and increases susceptibility to *Xanthomonas campestris pv. vesicatoria* infection. Transient expression of *CaPIK1* increases ROS generation and HR cell death.	10

Reference

1. Hong JK, Lee SC, Hwang BK. 2005. Activation of pepper basic PR-1 gene promoter during defense signaling to pathogen, abiotic and environmental stresses. Gene 356: 169-180.
2. Kim NH, Hwang BK. 2015. Pepper pathogenesis‐related protein 4c is a plasma membrane‐localized cysteine protease inhibitor that is required for plant cell death and defense signaling. The Plant Journal 81(1): 81-94.
3. Choi DS, Hwang IS, Hwang BK. 2012. Requirement of the cytosolic interaction between PATHOGENESIS-RELATED PROTEIN10 and LEUCINE-RICH REPEAT PROTEIN1 for cell death and defense signaling in pepper. The Plant Cell 24(4): 1675-1690.
4. Lee S, Hwang B. 2003. Identification of the pepper SAR8. 2 gene as a molecular marker for pathogen infection, abiotic elicitors and environmental stresses in Capsicum annuum. Planta 216(3): 387-396.
5. Lee SC, Hwang IS, Choi HW, Hwang BK. 2008. Involvement of the pepper antimicrobial protein CaAMP1 gene in broad spectrum disease resistance. Plant physiology 148(2): 1004-1020.
6. Kim NH, Lee DH, Choi DS, Hwang BK. 2015. The pepper GNA-related lectin and PAN domain protein gene, CaGLP1, is required for plant cell death and defense signaling during bacterial infection. Plant Science 241: 307-315.
7. Jung HW, Hwang BK. 2007. The leucine‐rich repeat (LRR) protein, CaLRR1, interacts with the hypersensitive induced reaction (HIR) protein, CaHIR1, and suppresses cell death induced by the CaHIR1 protein. Molecular plant pathology 8(4): 503-514.
8. Hong JK, Hwang IS, Hwang BK. 2017. Functional roles of the pepper leucine-rich repeat protein and its interactions with pathogenesis-related and hypersensitive-induced proteins in plant cell death and immunity. Planta 246(3): 351-364.
9. Kim DS, Hwang BK. 2014. An important role of the pepper phenylalanine ammonia-lyase gene (PAL1) in salicylic acid-dependent signalling of the defence response to microbial pathogens. Journal of experimental botany 65(9): 2295-2306.

10. Kim DS, Hwang BK. 2011. The pepper receptor - like cytoplasmic protein kinase CaPIK1 is involved in plant signaling of defense and cell - death responses. The Plant Journal 66(4): 642-655.
